# Unilateral Administration of Surface-Modified G1 and G4 PAMAM Dendrimers in Healthy Mice to Assess Dendrimer Migration in the Brain

**DOI:** 10.1021/acsami.4c09137

**Published:** 2024-07-31

**Authors:** Bhairavi Srinageshwar, Cassandra Thompson, Paulina Otero, Darren T. Story, Anna E. Wedster, Bethany MacDonald, Nikolas Munro, Sindhuja Koneru, Riley Crandall, Douglas Swanson, Ajit Sharma, Gary L. Dunbar, Julien Rossignol

**Affiliations:** † College of Medicine, Central Michigan University, Mount Pleasant, Michigan 48859, United States; ‡ Program of Neuroscience, Central Michigan University, Mount Pleasant, Michigan 48859, United States; § Field Neurosciences Institute Laboratory for Restorative Neurology, Central Michigan University, Mount Pleasant, Michigan 48859, United States; ∥ Department of Chemistry and Biochemistry, Central Michigan University, Mount Pleasant, Michigan 48859, United States; ⊥ Department of Psychology, Central Michigan University, Mount Pleasant, Michigan 48859, United States; □ Department of Psychology, Saginaw Valley State University, University Center, Michigan 48710, United States

**Keywords:** PAMAM dendrimers, brain clearance, nanoparticle, migration, cellular uptake, dendrimer transport

## Abstract

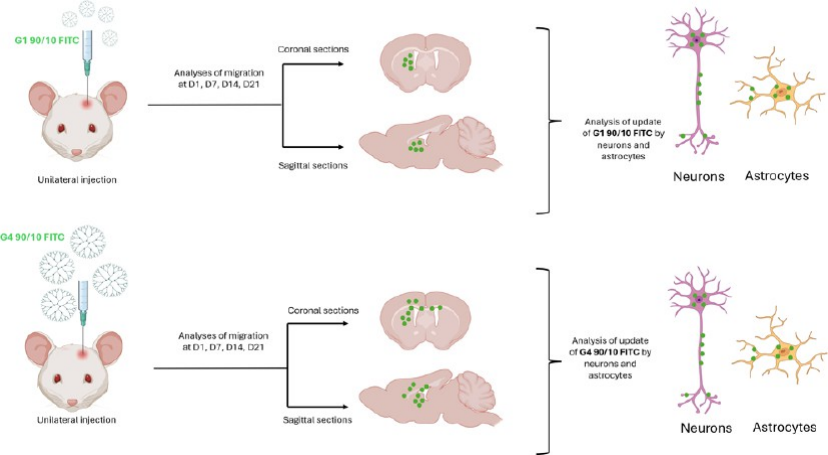

Polyamidoamine (PAMAM) dendrimers are nanoparticles that have a wide scope in the field of biomedicine. Previous evidence shows that the generation 4 (G4) dendrimers with a 100% amine surface (G4-NH_2_) are highly toxic to cells *in vitro* and *in vivo* due to their positively charged amine groups. To reduce the toxicity, we modified the surface of the dendrimers to have more neutral functional groups, with 10% of the surface covered with −NH_2_ and 90% of the surface covered with hydroxyl groups (−OH; G4–90/10). Our previous *in vitro* data show that these modified dendrimers are taken up by cells, neurons, and different types of stem cells *in vitro* and neurons and glial cells *in vivo*. The toxicity assay shows that these modified dendrimers are less toxic compared with G4-NH2 dendrimers. Moreover, prolonged dendrimer exposure (G1–90/10 and G4–90/10), up to 3 weeks following unilateral intrastriatal injections into the striatum of mice, showed that dendrimers have the tendency to migrate within the brain via corpus callosum at different rates depending on their size. We also found that there is a difference in migration between the G1 and G4 dendrimers based on their size differences. The G4 dendrimers migrate in the anterior and posterior directions as well as more laterally from the site of injection in the striatum compared to the G1 dendrimers. Moreover, the G4 dendrimers have unique projections from the site of injection to the cortical areas.

## 1 Introduction

Nanotechnology provides an avenue for improving efficacy and achieving efficient drug delivery in biological systems. Delivering drugs and therapeutics to the brain is even more challenging due to the presence of the blood–brain barrier (BBB). Overcoming this barrier to deliver drugs to the brain is a major first step toward treating neurological diseases by circumventing the need for invasive brain surgeries. Nanoparticle-based drug delivery systems can also help to deliver therapeutics to a targeted brain region, thereby reducing adverse effects due to nonspecific drug delivery.^1,2^

Though there are a number of platforms available to deliver cargo to the brain such as viral vectors, liposomes, carbon nanotubes and carbon sots, dendrimers, and micelles, only a few of them can cross the BBB and deliver drugs and therapeutics to the brain efficiently.^3^ The stability, solubility, and the ADME (absorption, distribution, metabolism, and excretion/clearance) properties of the nanodelivery system depend on many critical characteristics of the nanoparticle such as its overall size, potential toxicity, molecular weight, ζ-potential, and drug encapsulation and release efficacy.^4,5^

We have focused on using in-house synthesized polyamidoamine (PAMAM) dendrimer nanomolecules to deliver drugs and biomolecules to cells *in vitro* and to the brain *in vivo*. We have synthesized PAMAM (polyamidoamine) dendrimers of different sizes, surface groups, and cores to minimize toxicity and improve drug delivery efficacy. We tested these dendrimers in different cell models as well as in animal models. We have published data addressing toxicity^6−8^ and have shown that our surface-modified dendrimers (with 10% of the surface covered with −NH_2_ and 90% of the surface covered with hydroxyl groups −OH; G4–90/10 dendrimers used in this paper) were not toxic.

PAMAM dendrimers are nanomolecules ranging from 1 to 10 nm in diameter. The three components of the dendrimer are (1) a core, (2) generations (G), and (3) surface groups. We have described and reviewed PAMAM dendrimers previously, and our studies have shown that surface-modified dendrimers with 90% hydroxyl (−OH) and 10% amines (−NH_2_), known as G4–90/10 are safe *in vitro* and *in vivo*. We have also shown that surface-modified dendrimers have the ability to cross the blood–brain barrier (BBB) and deliver large cargos, such as plasmid DNA, to brain cells.^6−8^ In addition to the G4–90/10 dendrimers, we also synthesized G1 PAMAM dendrimers with 90% of the surface composed of −OH and 10% of −NH_2_^6^ (known as G1–90/10).

One of our previous studies showed that fluorescein isothiocyanate (FITC)-labeled G4–90/10 dendrimers were able to migrate between hemispheres via the corpus callosum 1 week following intrastriatal injections. Histological analysis showed that glial cells took up the dendrimers and migrated across the corpus callosum.^6^ Expanding on our previous study, the present study further investigated the migration of differently sized fluorescently tagged PAMAM dendrimers following injection into the striatum of healthy C57BL/6J mice. Two different sizes of PAMAM dendrimers, i.e., G1 and G4 [ ∼1 and 4 nm in diameter with diaminobutane (DAB) core, respectively], were injected unilaterally into the striatum, and the tissue was analyzed at four different time points to investigate the extent of migration through the brain tissue. We also observed the cellular uptake *in vivo* to analyze which cell types took up the different sized PAMAM dendrimers.

The outcome of the study (1) highlights the extent of PAMAM dendrimer migration and clearance from the brain and (2) provides a basis for an alternate strategy for delivering dendrimers and large-sized therapeutic cargo throughout the brain following unilateral rather than bilateral injections for cases in which systemic injection is not a viable option.

## 2 Results and Discussion

### 2.1 PAMAM Dendrimer Synthesis

The PAMAM G1–90/10 and G4–90/10 dendrimers were successfully synthesized, labeled, and characterized using our previously described methods.^6^

### 2.2 Neuronal and Glia Cell Expression Following G4–90/10 Dendrimers Uptake In Vitro

**Figure 1:**
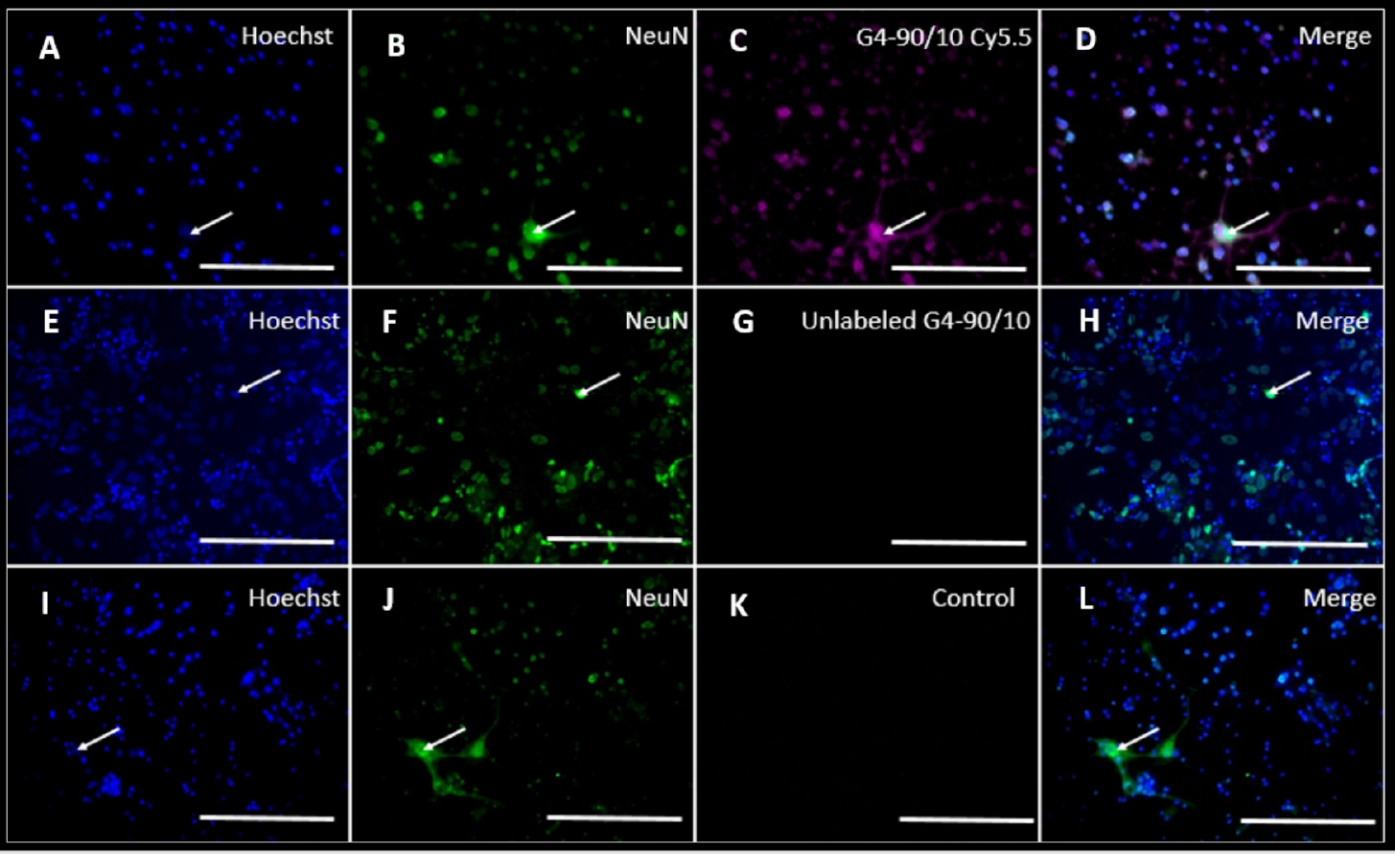
NeuN staining of PCC following dendrimers uptake: PCCs labeled with Hoechst 33342 stain (blue: A, E, I; arrow), neurons stained with NeuN (green: B, F, J; arrow), and G4–90/10 Cy5.5 stained with pink (C; arrow). (G, K) Unlabeled G4–90/10 dendrimers and the control cells, respectively. (D, H, L) Merge between the Hoechst, G4–90/10 Cy5.5, and the neurons (arrow). Colocalization in (D) shows that the neurons in PCC take up the dendrimers. Scale bar = 100 μm.

**Figure 2:**
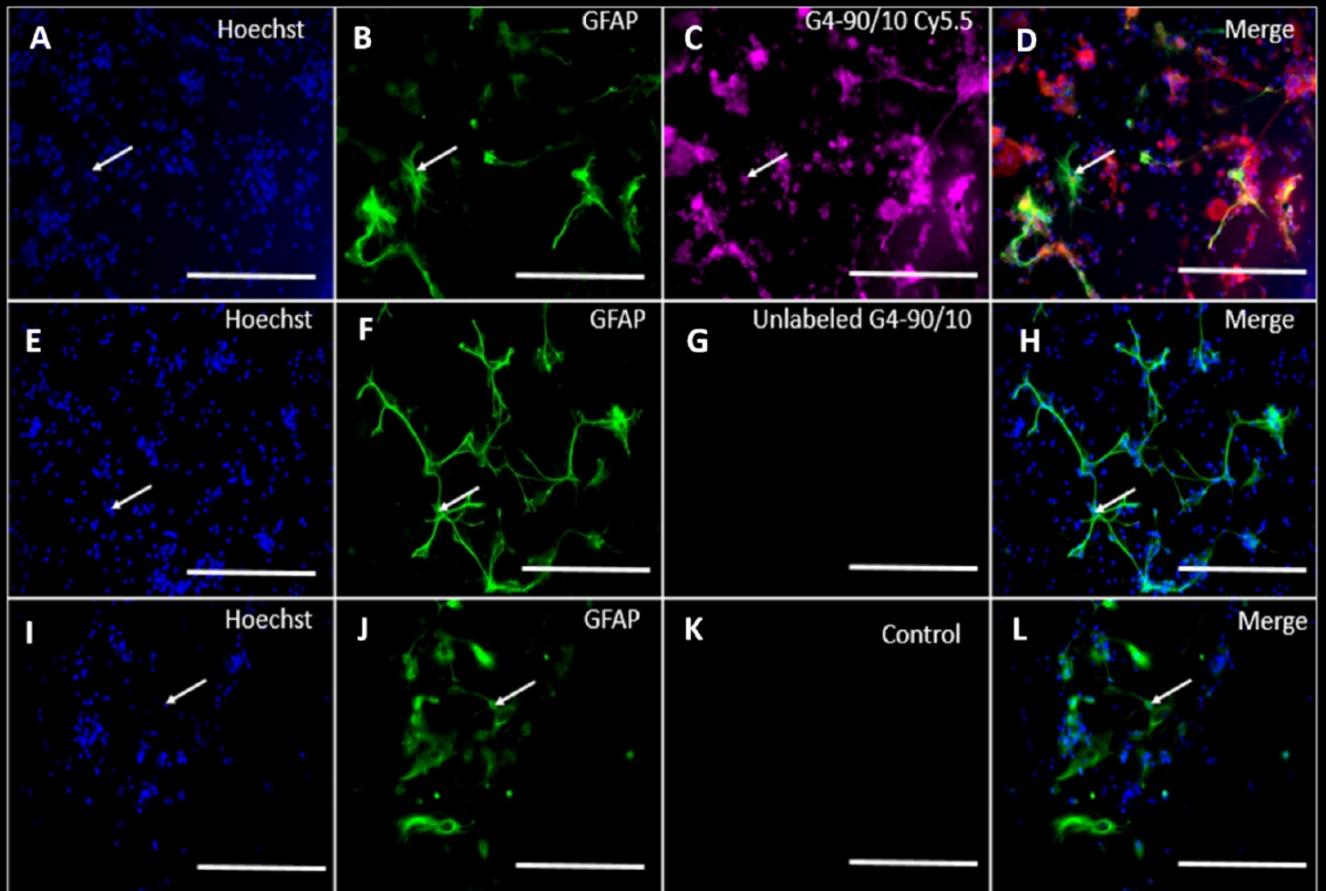
GFAP staining of PCC following dendrimers uptake: PCCs labeled with Hoechst 33342 stain (blue: A, E, I; arrow), glial cells stained with GFAP (green: B, F, J; arrow), and G4–90/10 Cy5.5 stained with pink (C; arrow). (G, K) Unlabeled G4–90/10 dendrimers and the control cells. (D, H, L) Merged between the Hoechst, G4–90/10 Cy5.5, and glial cells (arrow). Colocalization in (D) shows that the glial cells in PCC take up the dendrimers. Scale bar = 100 μm.

Following G4–90/10 Cy5.5 dendrimer (unlabeled and labeled with cyanine 5.5, Cy5.5) uptake by the primary cortical culture (PCC), the cells expressed NeuN (neuronal nuclei for mature neurons) and GFAP (glial fibrillary acidic protein for glial cells), showing that the dendrimers did not alter the development of neurons and glia compared to untreated cells (Figures 1 and 2). We previously showed that these dendrimers exhibited limited toxicity *in vitro*.^7^

### 2.3 Migration of G4–90/10 Dendrimers at 3 Weeks Following Injection

**Figure 3:**
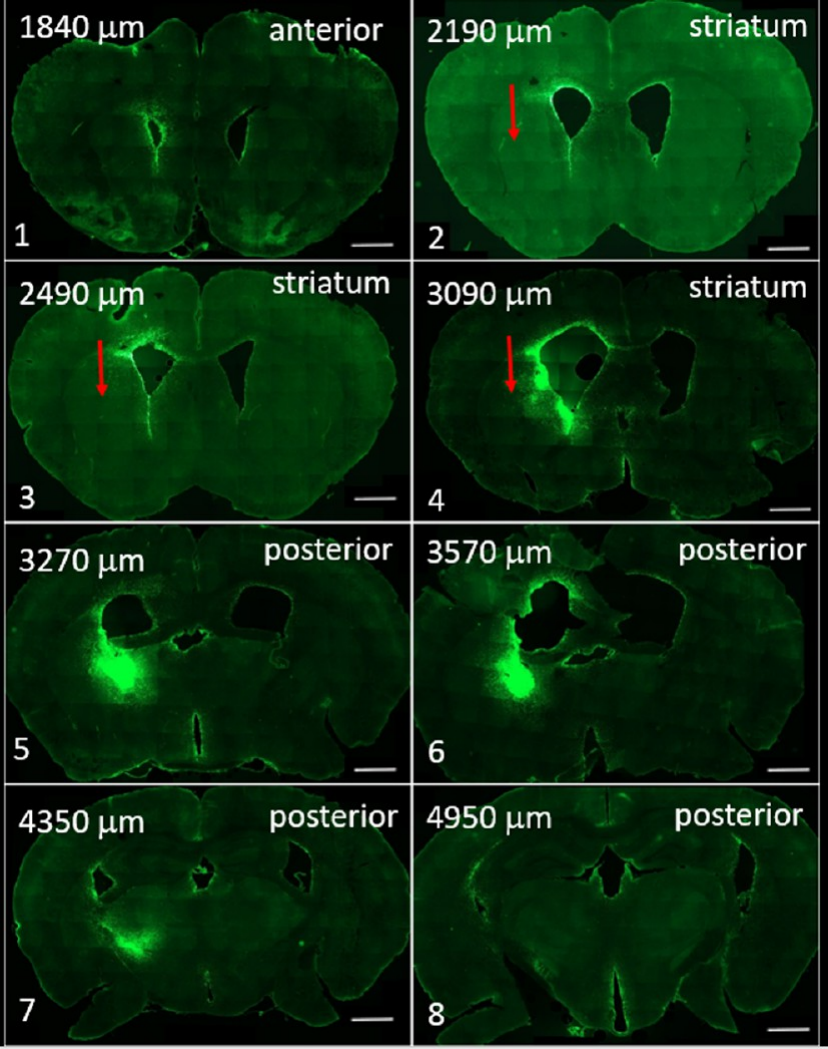
Coronal sections (from anterior to posterior marked from 1 to 8) of the mouse brain receiving G4–90/10-FITC dendrimers showing migration of dendrimers from anterior to posterior sections of the brain. Red arrows represent the site of dendrimer injection. Scale bar = 1000 μm.

Our results showed that the G4–90/10-FITC dendrimers migrated toward the anterior and posterior regions of the brain following 3 weeks of unilateral transplantation into the left striatum. Comparing the first and last slices of the brain where the dendrimers were seen, we found that the G4–90/10 dendrimers spread more anteriorly and posteriorly in the brain (Figure 3).

**Figure 4:**
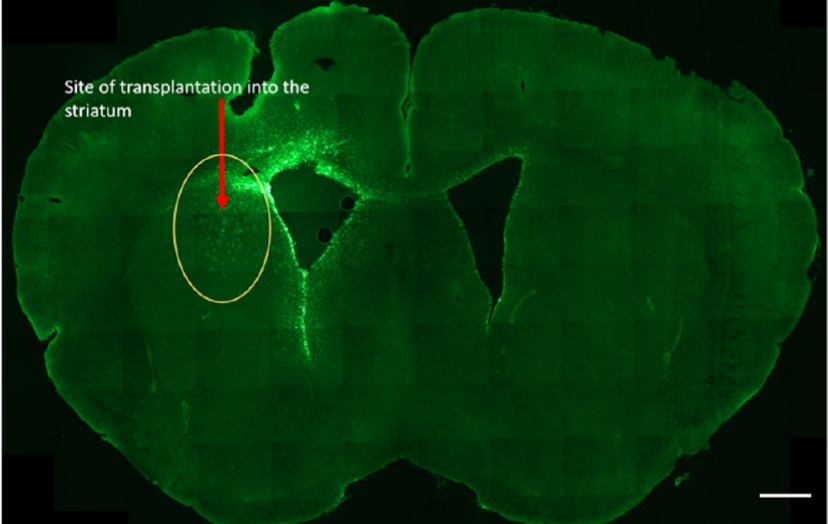
G4–90/10-FITC dendrimers seen in the left striatum 3 weeks following transplantation (circles and arrow). The dendrimers were also seen in the right striatum (arrow; near the lateral ventricles) and the corpus callosum suggesting that the dendrimers could have migrated across the corpus callosum, reaching the other hemisphere of the brain. Scale bar = 1000 μm.

**Figure 5:**
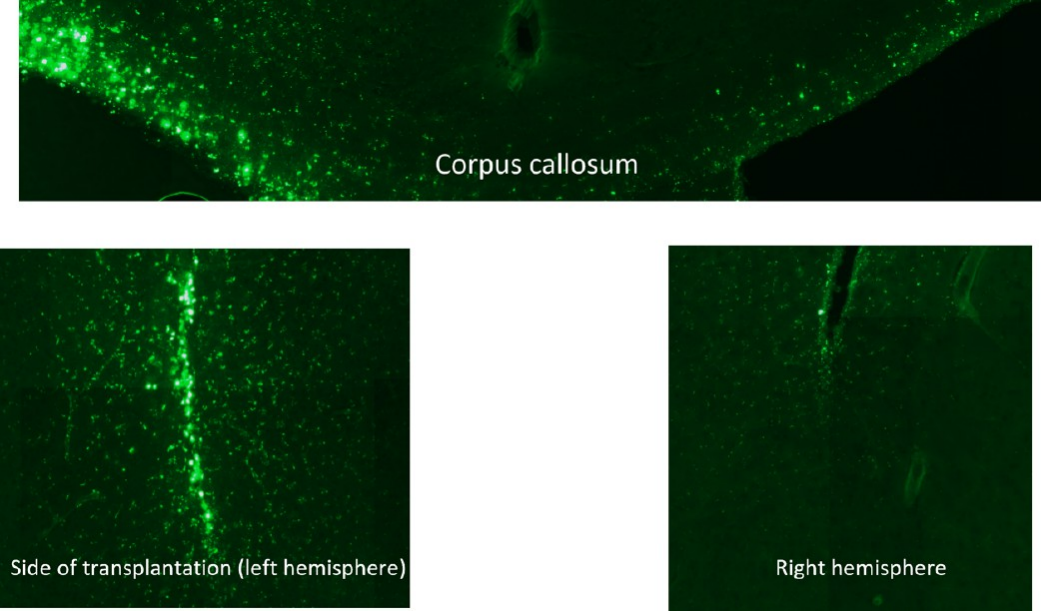
Zoomed images showing the G4–90/10-FITC dendrimers in the left hemisphere. The presence of these dendrimers in the corpus callosum shows that they have migrated to the right hemisphere, which did not receive dendrimer injection.

**Figure 6:**
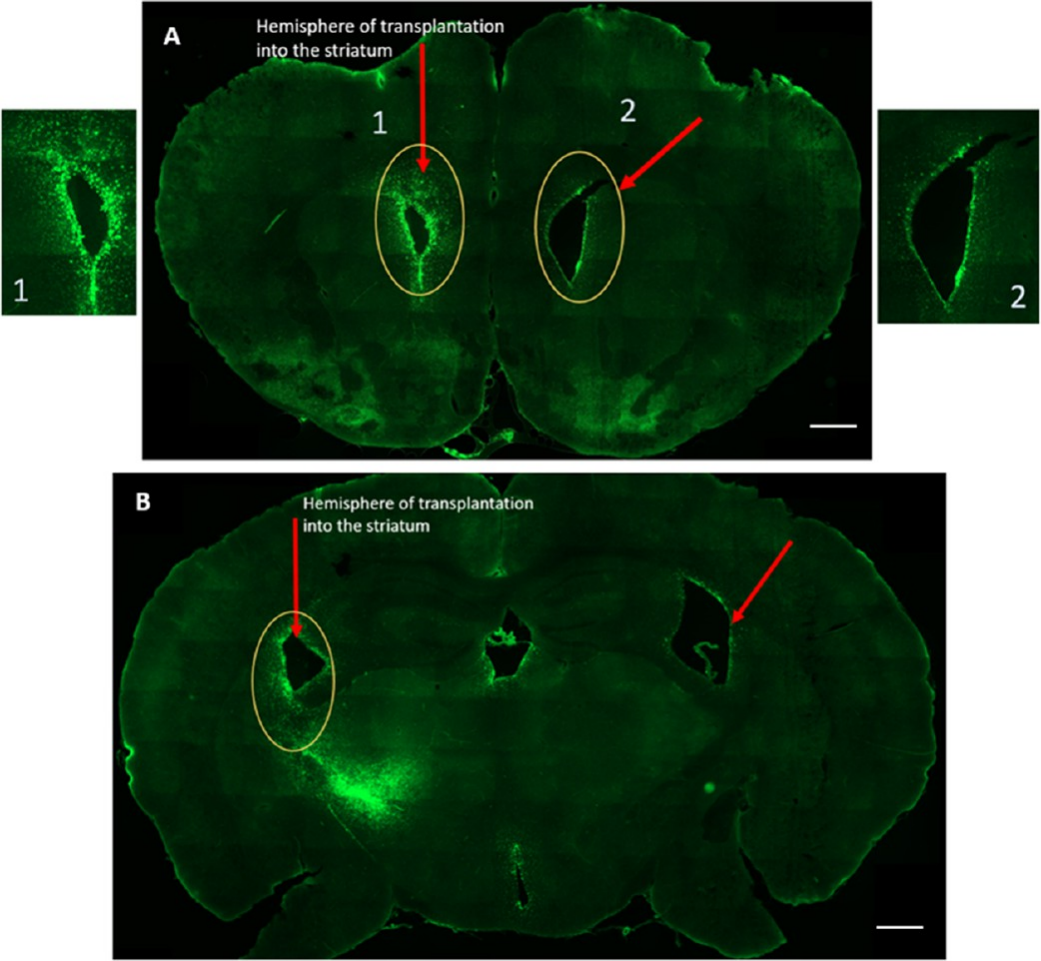
Presence of dendrimers in both the anterior (A) and posterior (B) regions of the brain: The G4–90/10-FITC dendrimers are seen in both hemispheres of the anterior region of the brain following transplantation into the left striatum. This shows that the dendrimers not only migrated between hemispheres but also migrated toward the anterior regions of the brain. Similar results were observed in the posterior region of the brain following transplantation into the left striatum. This shows that the dendrimers not only migrated between hemispheres but also migrated toward the posterior regions of the brain. Scale bar = 1000 μm.

Moreover, in addition to spreading more anteriorly and posteriorly, at 3 weeks following injection, the G4–90/10 FITC dendrimers also migrated across the corpus callosum and were seen around the ventricles in the right, contralateral, hemisphere (Figures 4–6).

**Figure 7:**
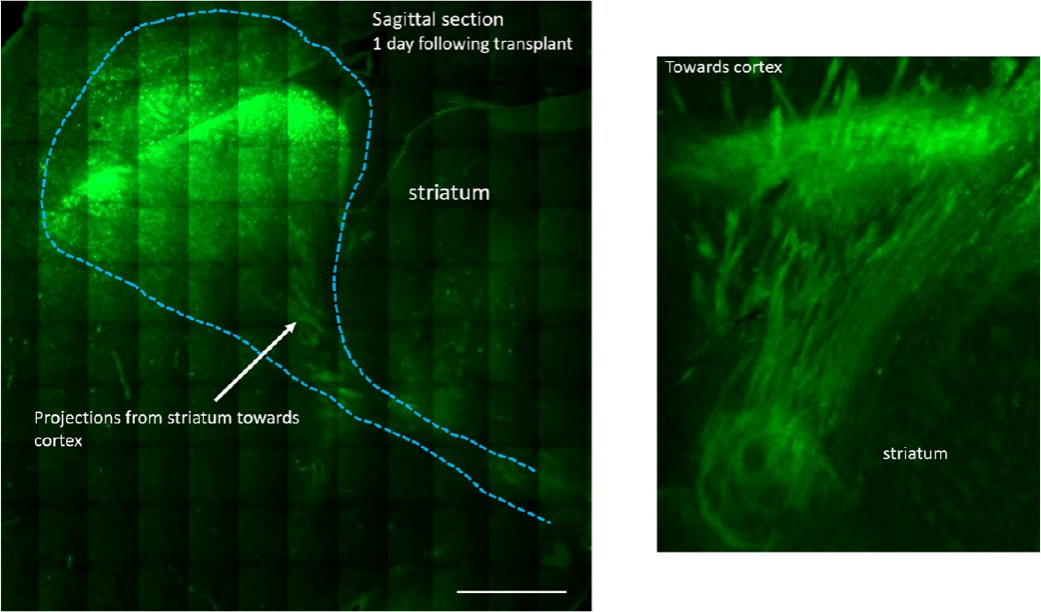
G4–90/10 dendrimers projecting from striatum toward the cortex: Sagittal sections of the brain receiving unilateral injections of the G4–90/10-FITC dendrimers showed the projects of the dendrimers from the striatum toward the cortical regions. These projections were not found in the brain that had received G1–90/10 FITC dendrimers. Scale bar = 1000 μm.

We also found that the G4–90/10-FITC dendrimers were projecting from the site of injection (striatum) toward the cortex 24 h following transplantation (Figure 7).

### 2.4 Migration of G1–90/10 Dendrimers from at 3 Weeks Following Injection

**Figure 8:**
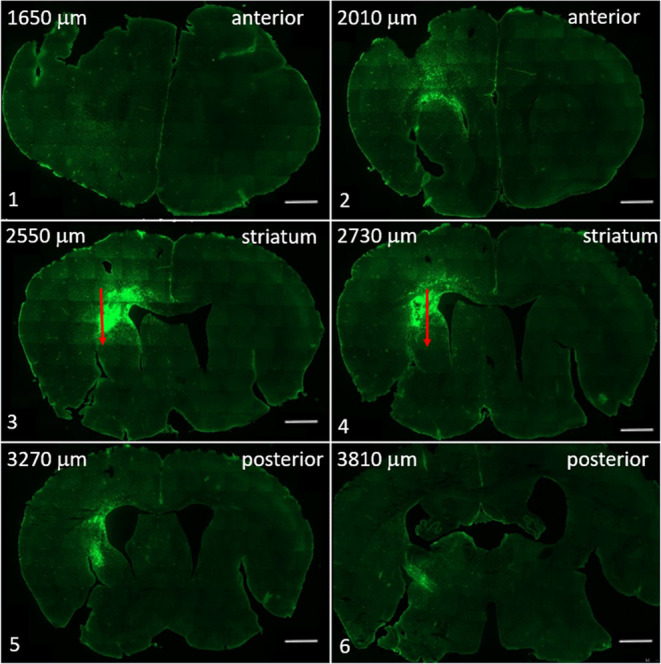
Coronal sections (from anterior to posterior marked from 1 to 6) of the mouse brain receiving G1–90/10-FITC dendrimers showing migration of dendrimers from anterior to posterior sections of the brain. Red arrows represent the site of dendrimer injection. Scale bar = 1000 μm.

Unlike G4–90/10-FITC dendrimers, G1–90/10-FITC dendrimers were not seen on the right hemisphere, showing that these dendrimers did not migrate across the corpus callosum (Figure 8).

**Figure 9:**
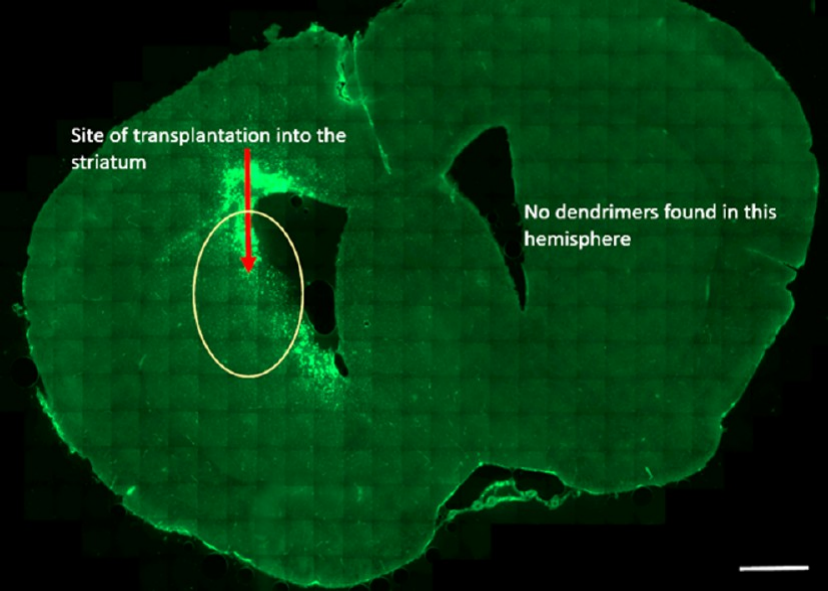
Presence of G1–90/10 dendrimers in the anterior region of the brain: The G1–90/10-FITC dendrimers are seen in the left hemisphere alone in the anterior region of the brain following transplantation into the left striatum. There are no dendrimers seen on the right hemisphere. Scale bar = 1000 μm.

**Figure 10:**
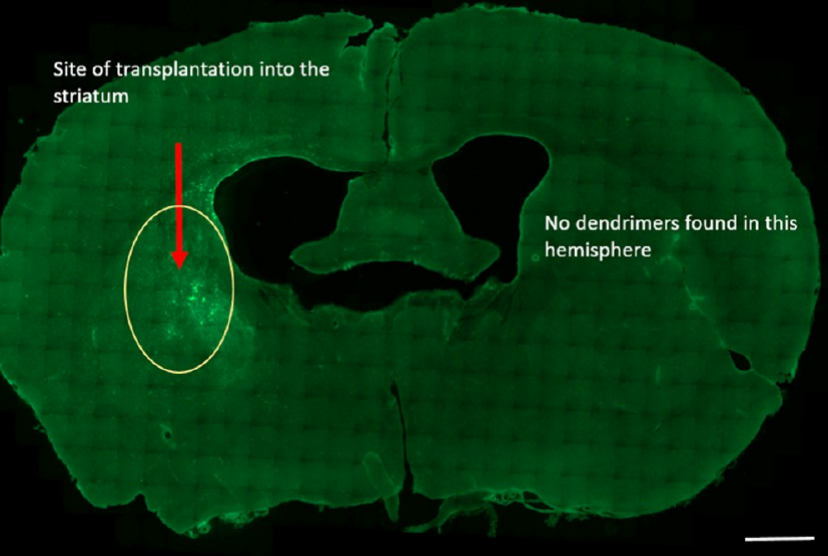
Presence of G1–90/10 dendrimers in the posterior region of the brain: The G1–90/10-FITC dendrimers are seen in the left hemisphere alone in the posterior region of the brain following transplantation into the left striatum. There are no dendrimers seen on the right hemisphere. Scale bar = 1000 μm.

However, our results showed that they were migrating toward the anterior and posterior regions (Figures 9 and 10) of the brain 3 weeks after unilateral transplantation into the left striatum.

**Figure 11:**
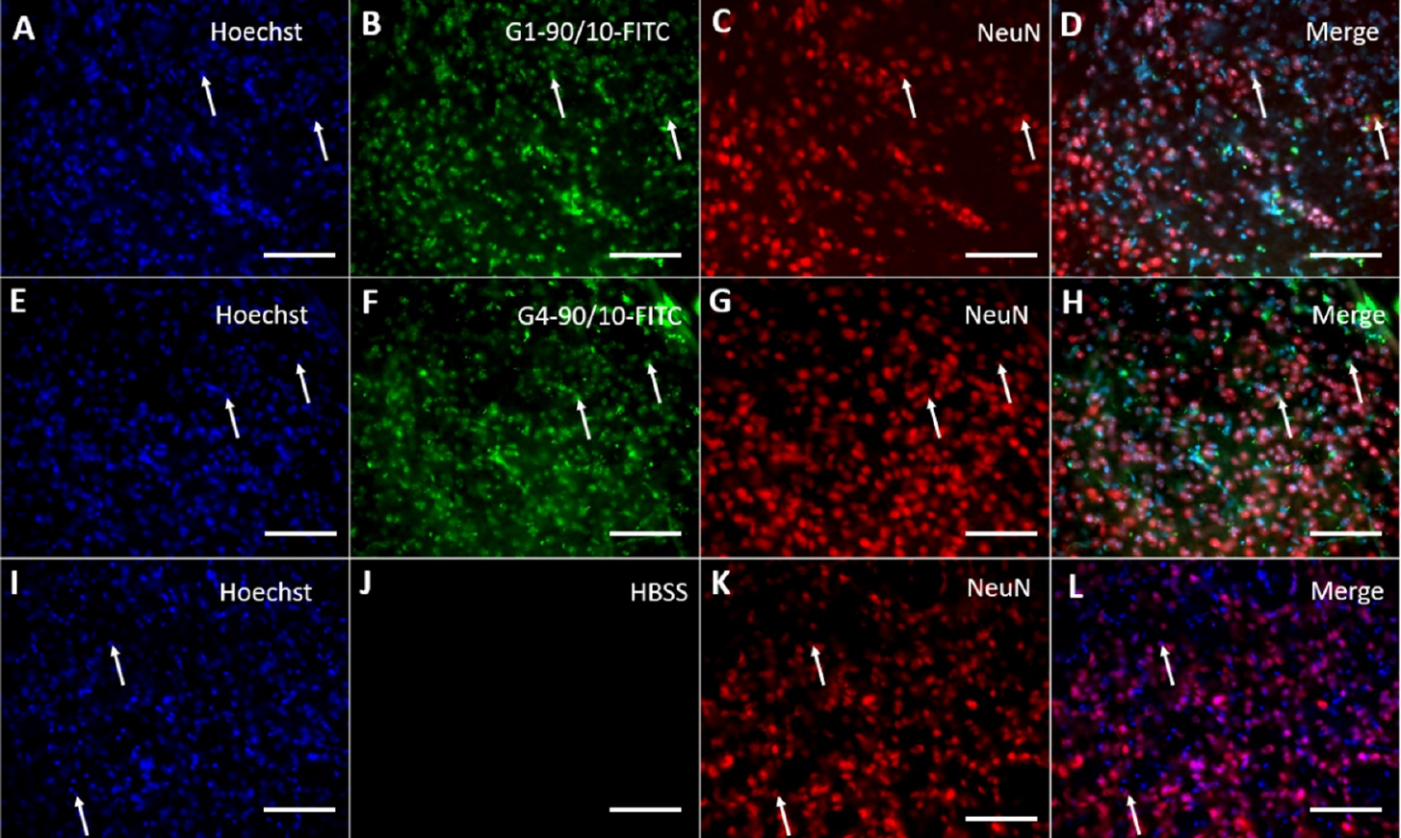
NeuN staining of the dendrimer brain tissue 3 weeks following their uptake: All of the cells in the brain were labeled with Hoechst (arrow; A, E, I). (B, F) G1–90/10-FITC and G4–90/10-FITC, respectively (arrow). (J) Hank’s balanced salt solution (HBSS). Neurons were stained using the NeuN antibody (arrow; C, G, K). Colocalization between G1–90/10-FITC and G4–90/10-FITC with NeuN (merges: D, H; arrow) shows that the neurons take up the dendrimers following their migration at 3 weeks. (L) Merge between control groups (Hoechst and NeuN). Scale bar = 100 μm.

**Figure 12:**
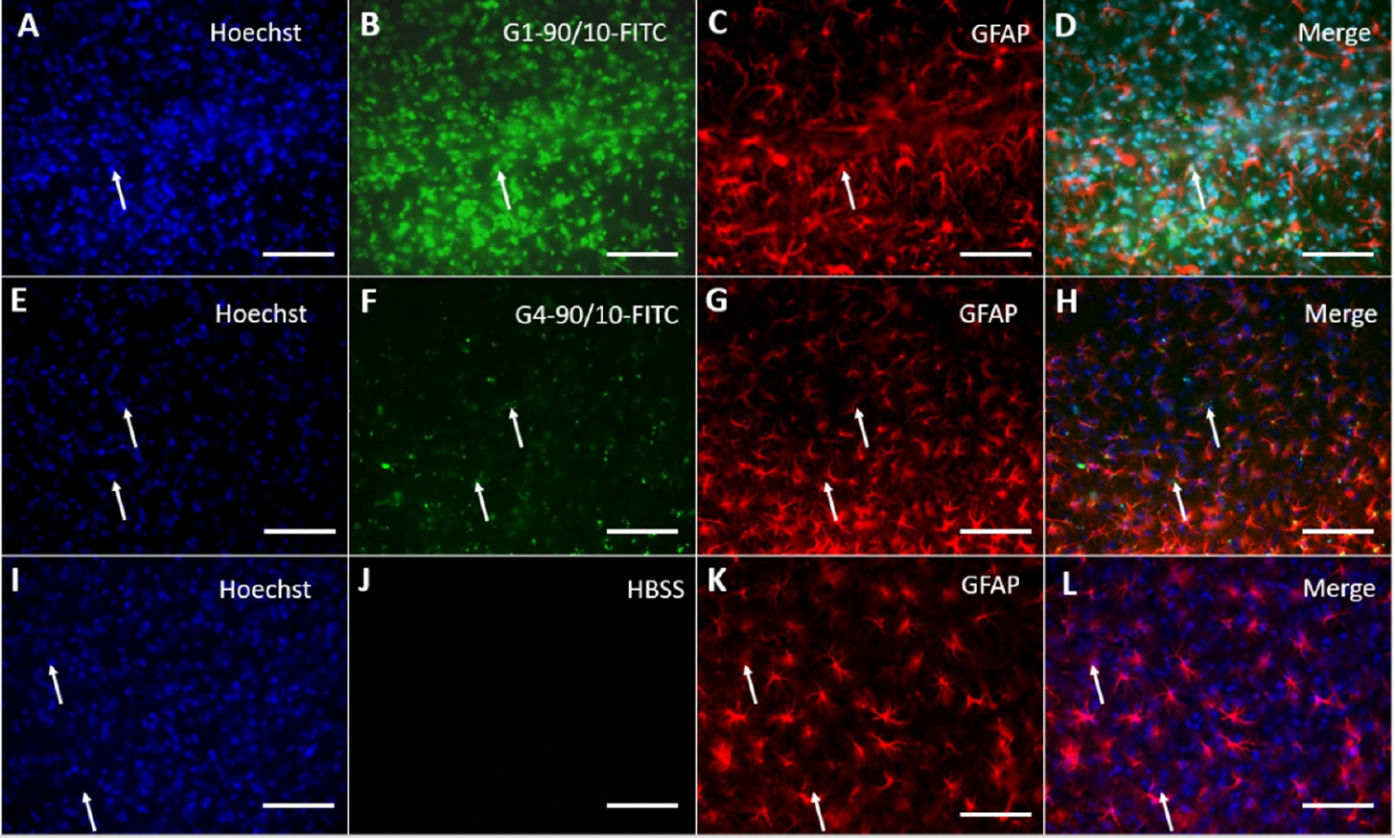
GFAP staining of the dendrimer brain tissue 3 weeks following their uptake: All of the cells in the brain were labeled with Hoechst (arrow; A, E, I). (B, F) G1–90/10-FITC and G4–90/10-FITC, respectively (arrow). (J) HBSS. Glial cells were stained using GFAP antibody (arrow; C, G, K). Colocalization between G1–90/10-FITC and G4–90/10-FITC with GFAP (merge: D, H; arrow) shows that the dendrimers are taken up by the glial cells following their migration at 3 weeks. (L) Merging between control groups (Hoechst and GFAP). Scale bar = 100 μm.

Following their migration in the brain, the dendrimers were found to be colocalized with neurons (NeuN stain) and glia (GFAP stain), demonstrating that the dendrimers are uptaken by multiple cell types in the brain (Figures 11 and 12).

**Figure 13:**
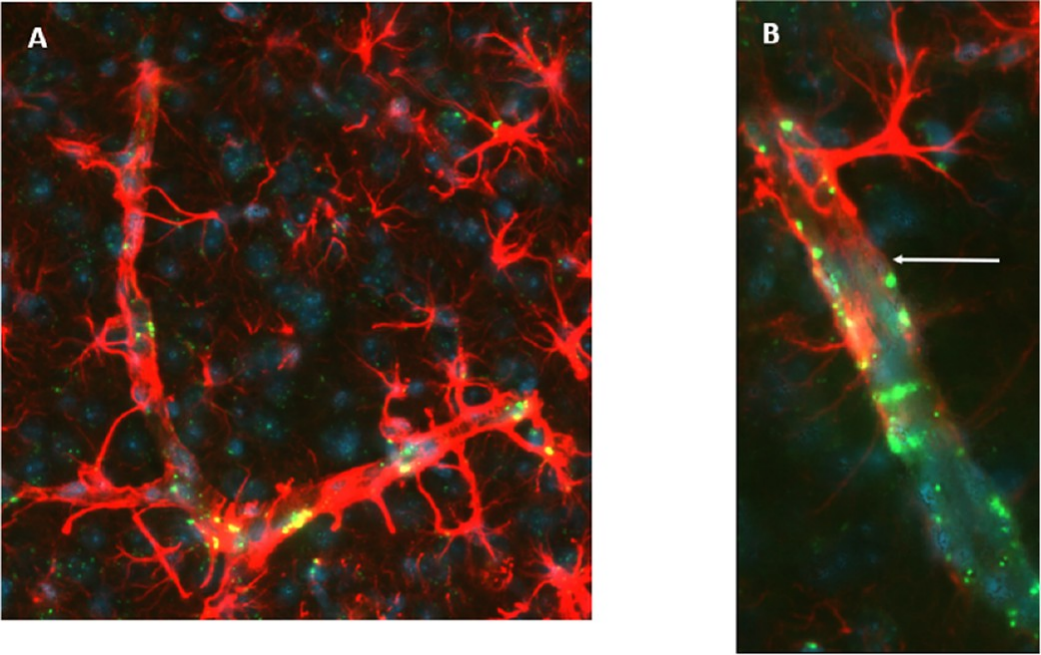
Presence of dendrimers in blood vessels: The dendrimers were also seen in the blood vessels 3 weeks (arrow) following transplantation (A, B). This may suggest that the dendrimers could be transported along the blood vessel from one region of the brain to the other, showing “migratory” properties.

Moreover, these G4–90/10-FITC dendrimers were also observed in the blood vessels, as shown below (Figure 13).

The results of this study and previous studies indicate that the G4–90/10 dendrimers are much safer than G4 amine-terminated dendrimers due to a 10-fold reduction in their amine surface. These safe, biocompatible PAMAM mixed-surface dendrimers are taken up *in vitro* by various cell types and are non-toxic. *In vivo*, intrastriatal injections of these dendrimers in healthy mice resulted in migration of these dendrimers in the brain. Further investigation revealed that following unilateral injections into the striatum of the left hemisphere, using different sized dendrimers such as G1–90/10 and G4–90/10, size-dependent migration was observed. The G4–90/10 dendrimers were found in both hemispheres as well as in the most anterior and posterior regions of the brain, showing that following their injection into the striatum, they were able to migrate throughout the brain. In contrast, G1–90/10 dendrimers were found only in the left hemisphere. However, they were present in both the anterior and posterior regions of that hemisphere, showing that following their injection into the striatum, they were able to migrate anterior to posterior within the hemisphere of injection. The difference in migration could be due to (1) the size difference in the dendrimers, (2) the difference in the metabolism and/or excretion rate of each of the dendrimers, or (3) size-dependent degradation of G1 and G4 dendrimers. Since the G1 dendrimers are smaller than the G4 dendrimers, there is a high possibility that all of the G1 dendrimers were taken up by the cells near the transplantation site, and no free dendrimers were left for migration to the other hemisphere. However, the G4 dendrimers, being relatively larger, can migrate and reach more cells since they were not all taken up by the cells at the transplantation site.

This was further confirmed using the GFAP marker, which showed that the dendrimers colocalized with astrocytes, which could further explain migration. Moreover, the dendrimers were also in the blood vessels, and both G1 and G4 dendrimers were colocalized with the epithelial cells surrounding blood vessels. This shows that the dendrimers could move along the blood vessels in the brain to different regions. However, the reason that the G1 dendrimers alone were not found in the other hemisphere needs further investigation. The dendrimers surrounding the lateral, third, and fourth ventricles suggest that the dendrimers could be in circulation with cerebrospinal fluid (CSF). This may also explain how these dendrimers migrate in the brain and possibly become eliminated over the course of time. However, this explanation may not be valid for the G1 dendrimers, as they were migrating only in one hemisphere. Longer time points than 3 weeks are required to investigate the biodistribution, migratory, and clearance properties of the differently sized dendrimers.

Finally, we observed unique projections of the G4–90/10 FITC dendrimers from the striatum to the cortical regions. A previous study by Salegio and colleagues investigated the movement and biodistribution of different viral serotypes and nanoparticles (such as micelles and liposomes) in Sprague–Dawley rats and rhesus monkeys. The study outcome showed a difference in the migratory patterns of viruses and nanomaterials in the brain of rodent and nonhuman primates. The unique biodistribution, transportation, and migratory patterns were attributed to arterial blood pressure and cardiac cycle.^9,10^

Another major factor that is yet to be explored is the migratory properties of different nanomaterials in the diseased brain (such as Alzheimer’s disease and Parkinson’s disease) as the CSF flow and axonal transport are altered compared to a healthy brain.^11,12^

## 3 Conclusions

This study emphasizes the primary basis toward analyzing the migratory effects of PAMAM dendrimers in healthy brain, thereby opening avenues to study the effects of dendrimer transportation in the diseased brain.

## 4 Materials and Methods

### 4.1 Animals

A total of 72 male and female C57BL/6J mice were used in this study, including one pregnant female utilized for the extraction of PCC. All of the animal procedures followed the guidelines of the Institutional Animal Care and Use Committee (IACUC) of Central Michigan University (August 16, 2018, and was registered under the CMU IACUC protocol #18-23). All of the mice were housed in clear polycarbonate cages with a 12 h light/12 h dark cycle. Food and water were accessible to the mice *ad libitum*.

### 4.2 Different Sized PAMAM Dendrimer Synthesis

The G1–90/10 and G4–90/10 dendrimers were synthesized as previously described. The G1–90/10 dendrimers were labeled with FITC, and the G4–90/10 dendrimers were labeled with both FITC and Cy5.5. They were also characterized as described in our previous publication.^6^

### 4.3 In Vitro

#### 4.3.1 Characterization of the PCC Following G4–90/10 Dendrimer Uptake

Primary cortical cells were extracted from E18 embryos obtained from pregnant C57BL/6J mouse and maintained as previously described. The G4–90/10 dendrimers were administered to cells at a final concentration of 4 mg/mL, and their uptake by the PCC was assessed as previously described.^6^ Immunocytochemistry was then used to analyze dendrimer uptake by neurons and glial cells. The PCC were plated on coverslips precoated with 0.2 mg/mL poly-l-lysine (Sigma-Aldrich, St. Louis, MO). Cy5.5-labeled and unlabeled G4–90/10 were added to the PCC to a final concentration of 4 mg/mL and then incubated for 30 min. PCC without dendrimers were used as a control. Following incubation, the cells were washed with 0.01 M phosphate-buffered saline (PBS) and fixed with 4% paraformaldehyde (PFA; Sigma-Aldrich). To stain for neurons and glial cells, rabbit-antineuronal nuclei antibody (NeuN 1/5000; ab177487, Abcam, Cambridge, U.K.) and rabbit-antiglial fibrillary acidic protein antibody (GFAP 1/5000; ab7260, Abcam) were dissolved in 0.01 M PBS with 0.1% saponin and 0.02% sodium azide (Sigma-Aldrich). Cells were incubated with the primary antibody for 1 h, and then the cells were washed once with 0.01 M PBS. After washing, the secondary antibody (Alexa Fluor 488 goat anti-chicken Ig at 1:300 dilution; Thermo Fisher Scientific, Waltham, MA) was added to the cells and incubated for 1 h. The cells were then washed twice with 0.01 M PBS, and Hoechst 33342 (Thermo Fisher Scientific) was added at a concentration of 1:500 dissolved in 0.01 M PBS with 0.1% saponin and 0.02% sodium azide and then incubated for an additional 5 min. The cells were again washed twice with 0.01 M PBS, and coverslips were mounted on the slides. The cells were then imaged by using a Zeiss Axio Imager M1 microscope (Carl Zeiss AG).

### 4.4 In Vivo

#### 4.4.1 Animals and Groups

The animals were randomly divided into three groups: (1) G1–90/10 mice (*n* = 24), (2) G4–90/10 mice (*n* = 24), and (3) Hank’s balanced salt solution (HBSS) vehicle control mice (*n* = 24). Each group consists of four subgroups sacrificed at different time points: 24 h, 1 week, 2 weeks, and 3 weeks following dendrimer transplantation (Table 1).

**Table 1:** Animal and Groups

group	number of animals (subgroup)	route of administration	animal end points
HBSS	6	unilateral intracranial injection into the striatum	24 h following transplantation
	6		1 week following transplantation
	6		2 weeks following transplantation
	6		3 weeks following transplantation
G1–90/10-FITC	6	unilateral intracranial injection into the striatum	24 h following transplantation
	6		1 week following transplantation
	6		2 weeks following transplantation
	6		3 weeks following transplantation
G4–90/10-FITC	6	unilateral intracranial injection into the striatum	24 h following transplantation
	6		1 week following transplantation
	6		2 weeks following transplantation
	6		3 weeks following transplantation
total	72		

#### 4.4.2 Unilateral Intrastriatal Injections of G1–90/10-FITC and FITC-G4–90/10-FITC PAMAM Dendrimers

Male and female C57BL/6J mice were injected with 2 μL of either G1–90/10-FITC or G4–90/10-FITC dendrimers at a concentration of 10 mg/mL via unilateral intracranial injections into the striatum. Vehicle controls were injected with HBSS. The surgical procedures were the same as in our previous publication.^6^ Briefly, for unilateral injections, anesthetized mice were placed in ear bars and burr holes (0.5 mm) were made over the left neostriatum (coordinates from bregma: anterior/posterior +0.5 mm; medial/lateral ±1.75 mm; dorsal/ventral −2.5 mm). The animals were observed daily, and postoperative care was given for five consecutive days following injections.

#### 4.4.3 Euthanasia

The animals were sacrificed by cervical dislocation at 24 h, 1 week, 2 weeks, or 3 weeks following dendrimer injection. Brains were extracted and postfixed in 4% PFA (Sigma-Aldrich) for 48 h, after which the tissue was transferred to 10, 20, and 30% gradient sucrose solution, then frozen using 2-methylbutane (Sigma-Aldrich), and stored at −80 °C until further use.

#### 4.4.4 Histology

The tissue was sectioned in a cryostat at a 30 μm thickness. Three brains from each subgroup were sliced into coronal sections, and the other three brains from each subgroup were sliced into sagittal sections to analyze the migration of the dendrimers from medial to lateral and anterior to posterior, respectively. Sections at 210 μm intervals were analyzed to locate the G1–90/10-FITC or G4–90/10-FITC dendrimers and their migration in the brain at four different time points. To analyze the uptake and migration of the G1–90/10-FITC or G4–90/10-FITC dendrimers by neurons and glial cells, the tissue was stained using rabbit-anteneuronal nuclei antibody (NeuN 1/3000; ab177487, Abcam, Cambridge, U.K.) diluted in 0.01 M phosphate-buffered saline with 0.1% triton X-100 (Fluka Chemicals, Mexico City, Mexico) and rabbit-antiglial fibrillary acidic protein antibody (GFAP; 1/3000; ab7260 Abcam, Cambridge, U.K.) diluted in 0.01 M phosphate-buffered saline with 0.3% triton X-100 (Fluka Chemicals, Mexico City, Mexico). Cell nuclei were stained with Hoechst 33342 (Thermo Scientific) at a 1:1000 dilution. The images were analyzed for dendrimer migration and colocalization with neurons and glial cells using a Zeiss Axio Imager M1 microscope (Carl Zeiss AG).

## Abbreviations

ADME: absorption, distribution, metabolism, and excretion/clearance
NH2: amine group
BBB: blood–brain barrier
CSF: cerebrospinal fluid
Cy5.5: cyanine 5.5
DAB: diamino butane
FITC: fluorescein isothiocyanate
G1: generation 1
G4: generation 4
GFAP: glial fibrillary acidic protein
HBSS: Hank’s balanced salt solution
OH: hydroxyl group
NeuN: neuronal nuclei
PAMAM: polyamidoamine
PFA: paraformaldehyde
PBS: phosphate-buffered saline
PCC: primary cortical culture
